# Supplementary material for: The Difference in Cytotoxic Activity between Two Optical Isomers of Gelsemine from Gelsemium elegans Benth. on PC12 Cells
Source: Molecules. 2019 May 25;24(10):2004. doi: 10.3390/molecules24102004 (PMC6571982; doi:10.3390/molecules24102004)
Supplement: Supplementary file 1 [file molecules-24-02004-s001.pdf]

molecules

The Difference in Cytotoxic Activity between Two Optical Isomers of Gelsemine from Gelsemium elegans Benth. on PC12 Cells

Li Lin[1], Yan-Chun Liu[1,2], and Zhao-Ying Liu[1,2,*]

[1] College of Veterinary Medicine, Hunan Agricultural University, Changsha 410128, Hunan, China
[2] Hunan Engineering Technology Research Center of Veterinary Drugs, Hunan Agricultural University, Changsha 410128, Hunan, China
* Correspondence: liu_zhaoying@hunau.edu.cn

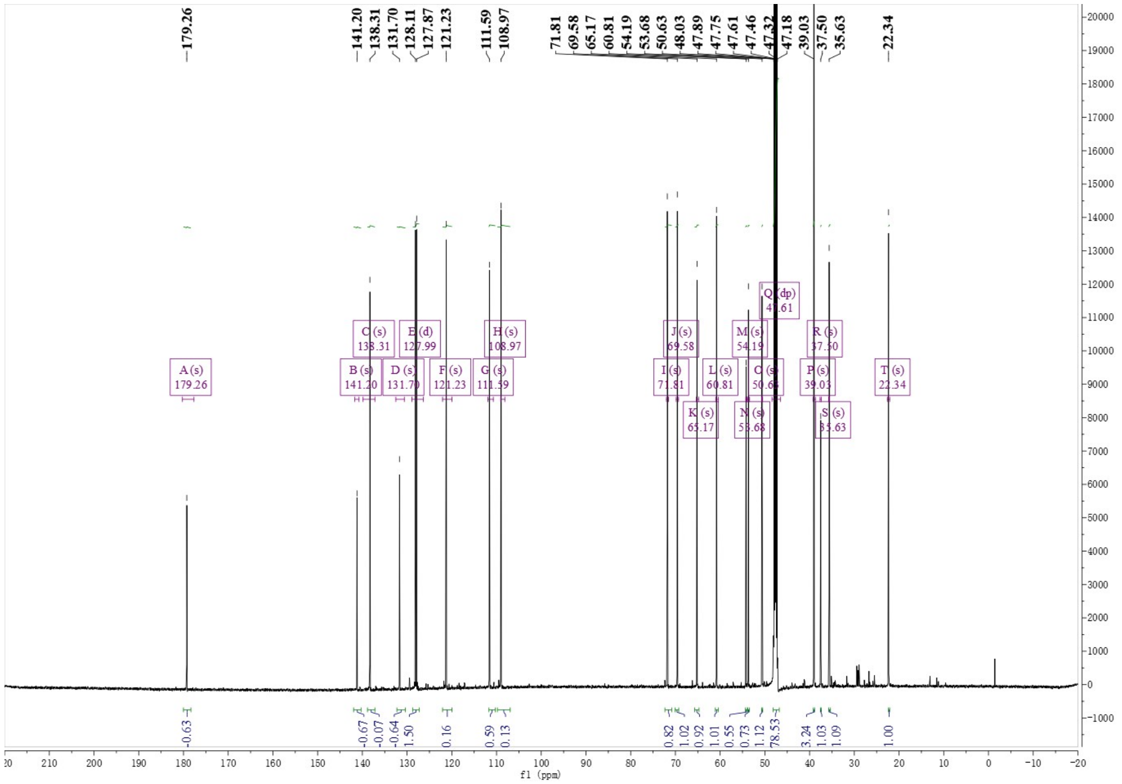

Figure S1. The ^{13}C NMR spectrum of compound **1**.

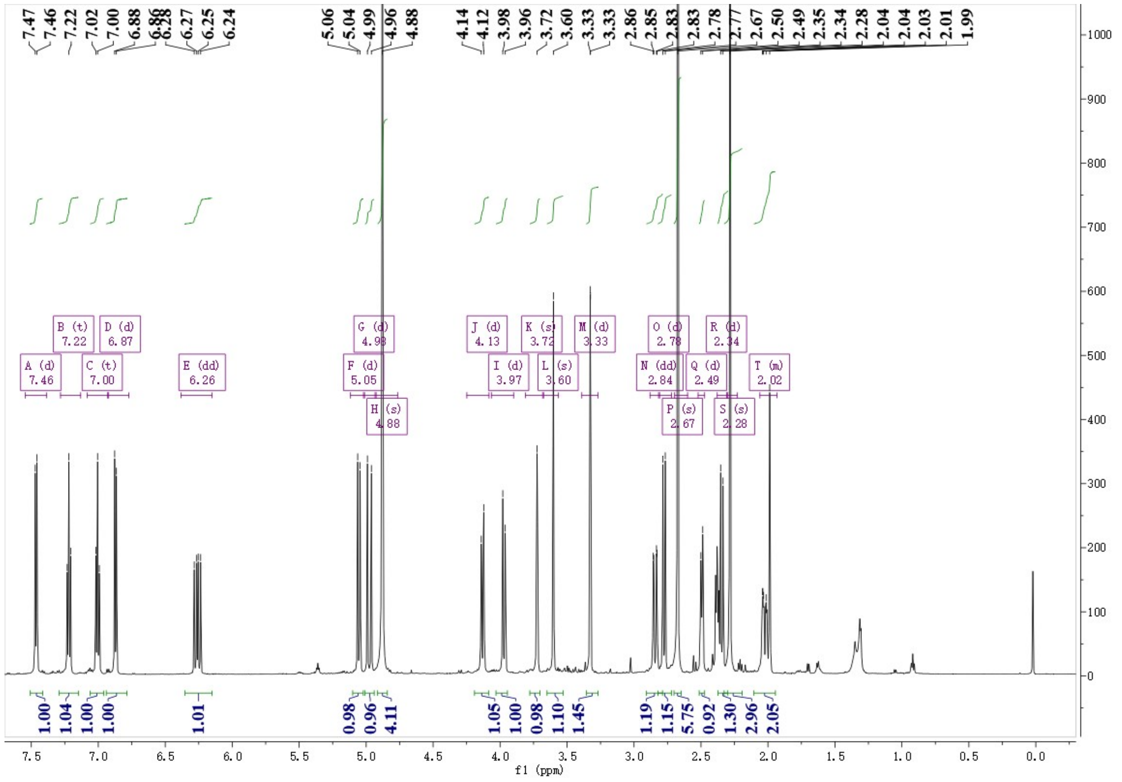

Figure S2. The ^{1}H NMR spectrum of compound **1**.

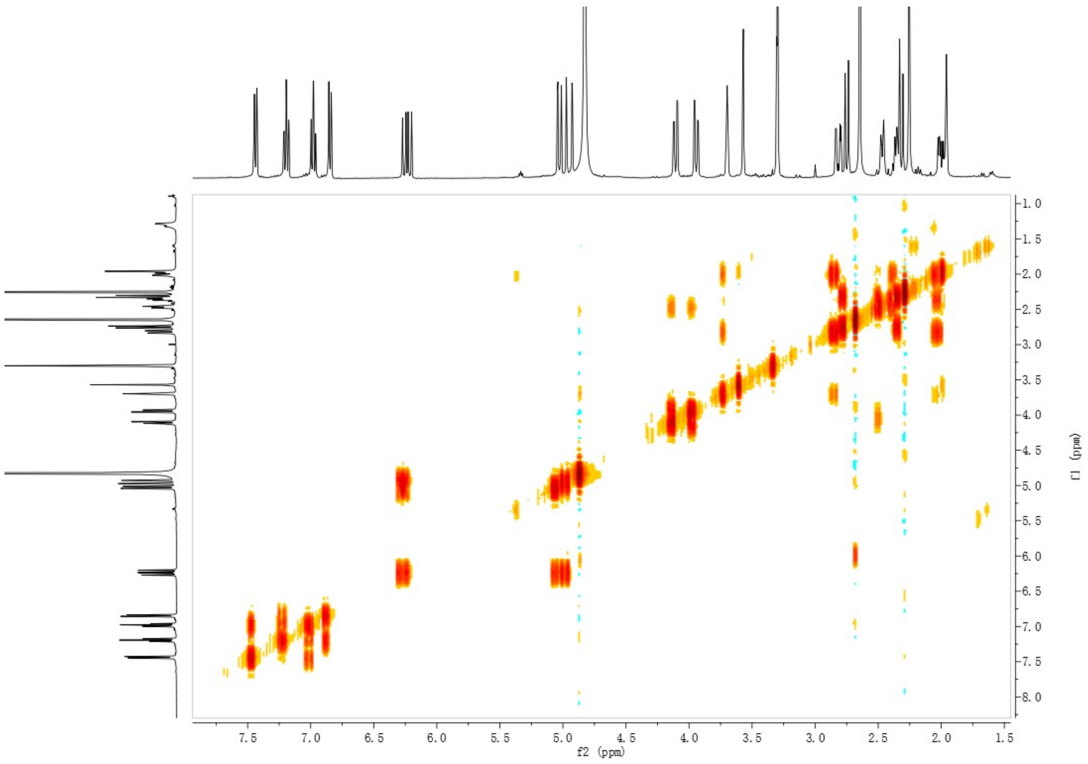

Figure S3. The ^{1}H-^{1}H COSY spectrum of compound **1**.

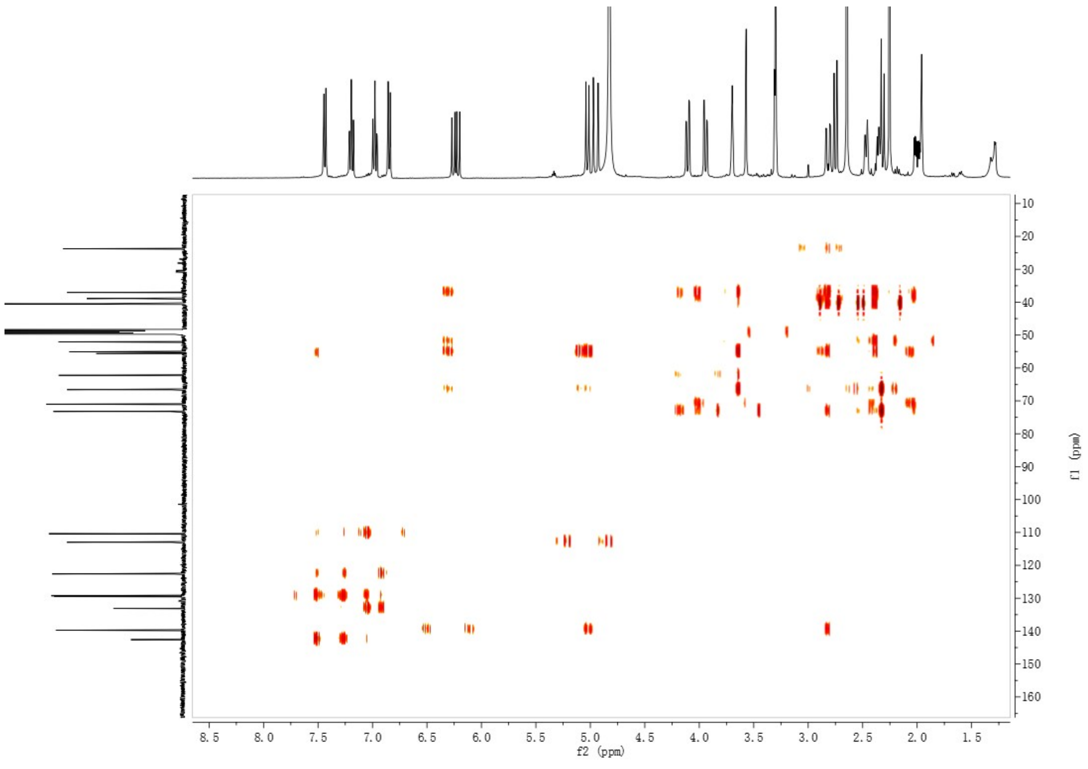

Figure S4. The HMBC spectrum of compound **1**.

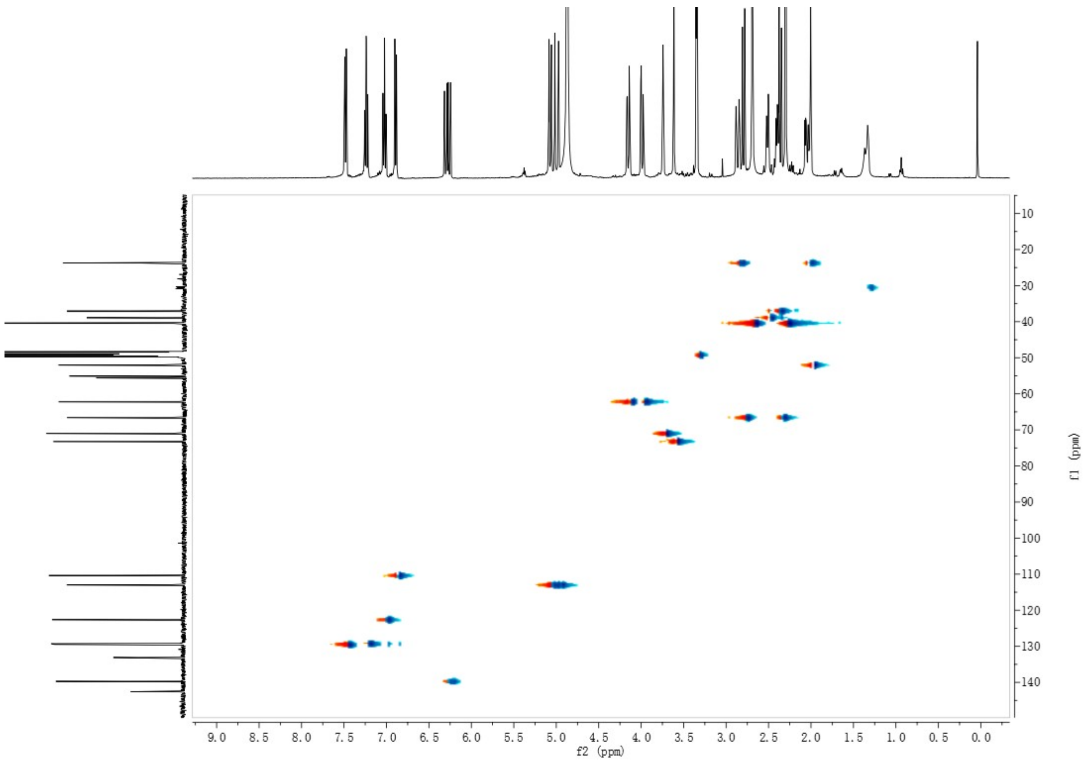

Figure S5. The HSQC spectrum of compound **1**.

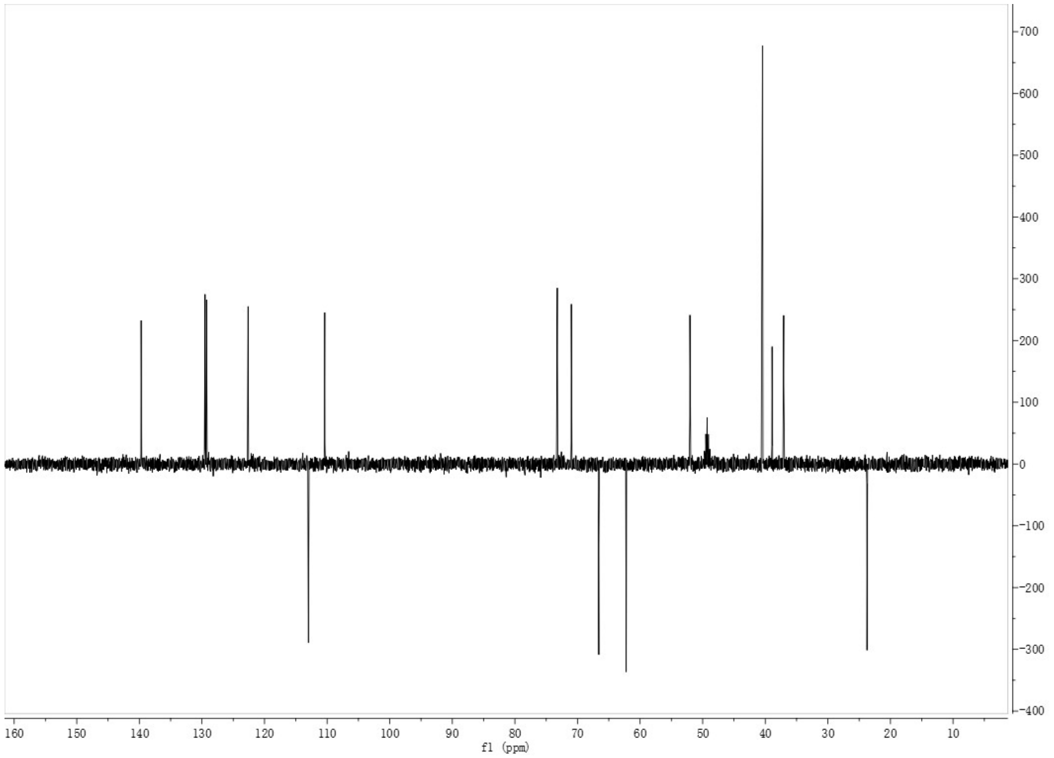

Figure S6. The DEPT135 spectrum of compound **1**.

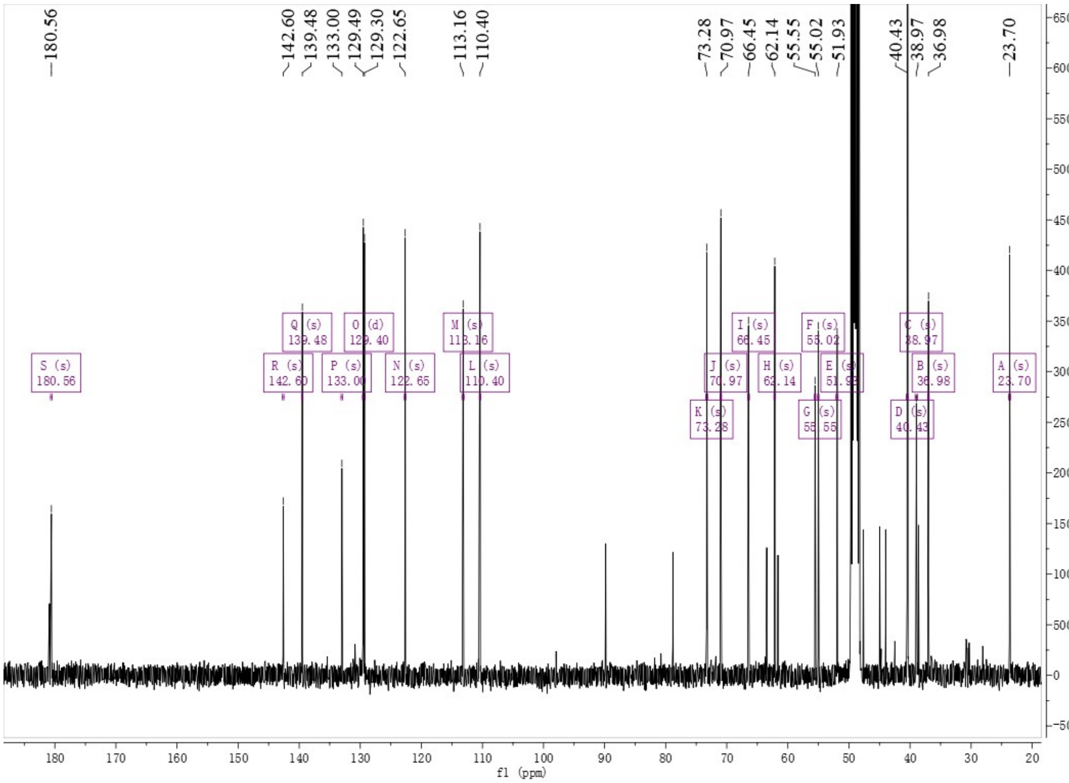

Figure S7. The ^{13}C NMR spectrum of compound **2**.

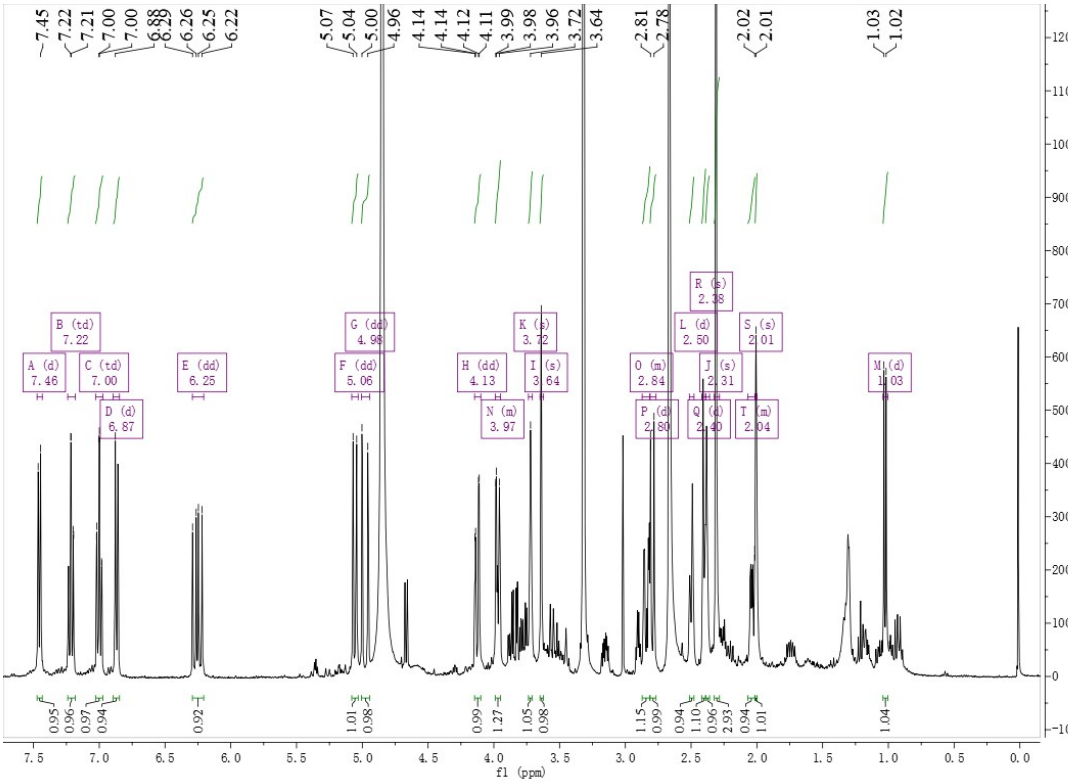

Figure S8. The ^{1}H NMR spectrum of compound **2**.

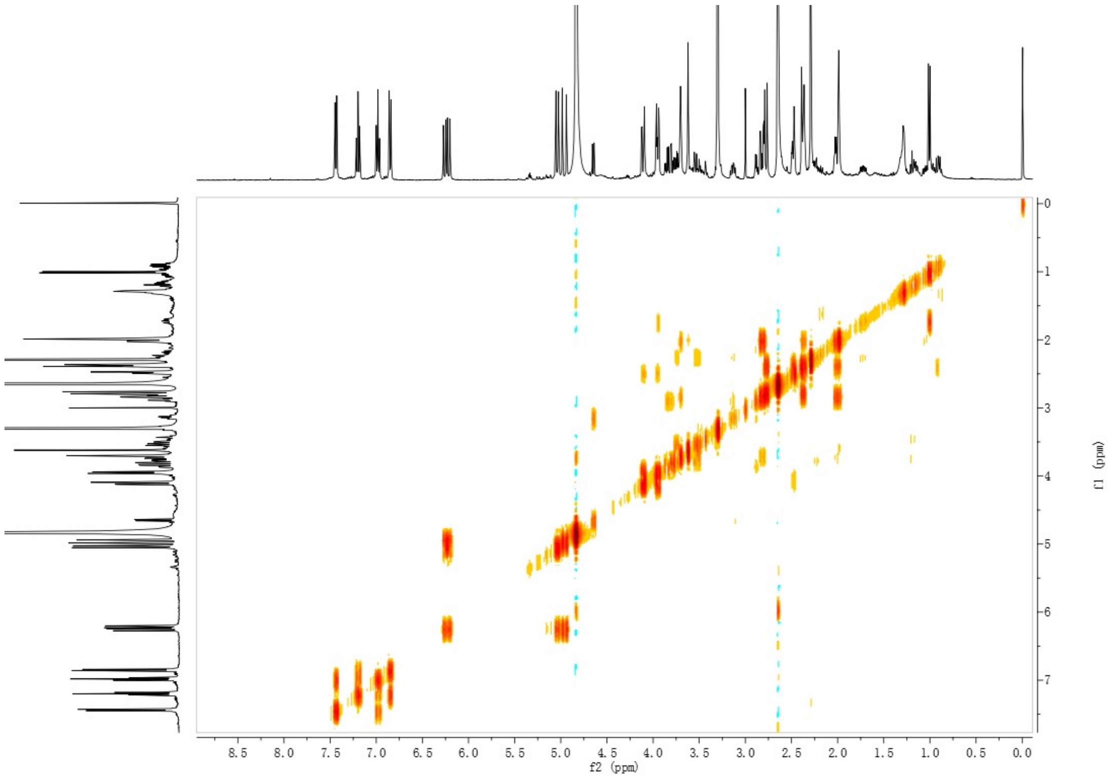

Figure S9. The ^{1}H-^{1}H COSY spectrum of compound **2**.

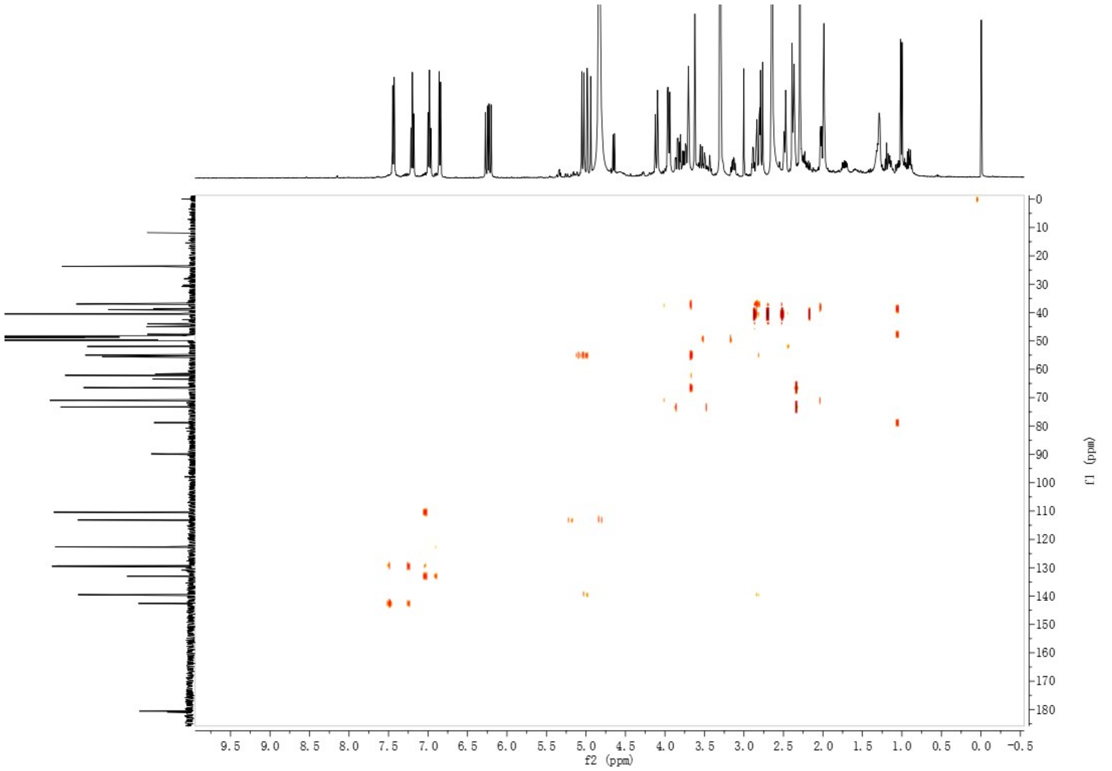

Figure S10. The HMBC spectrum of compound **2**.

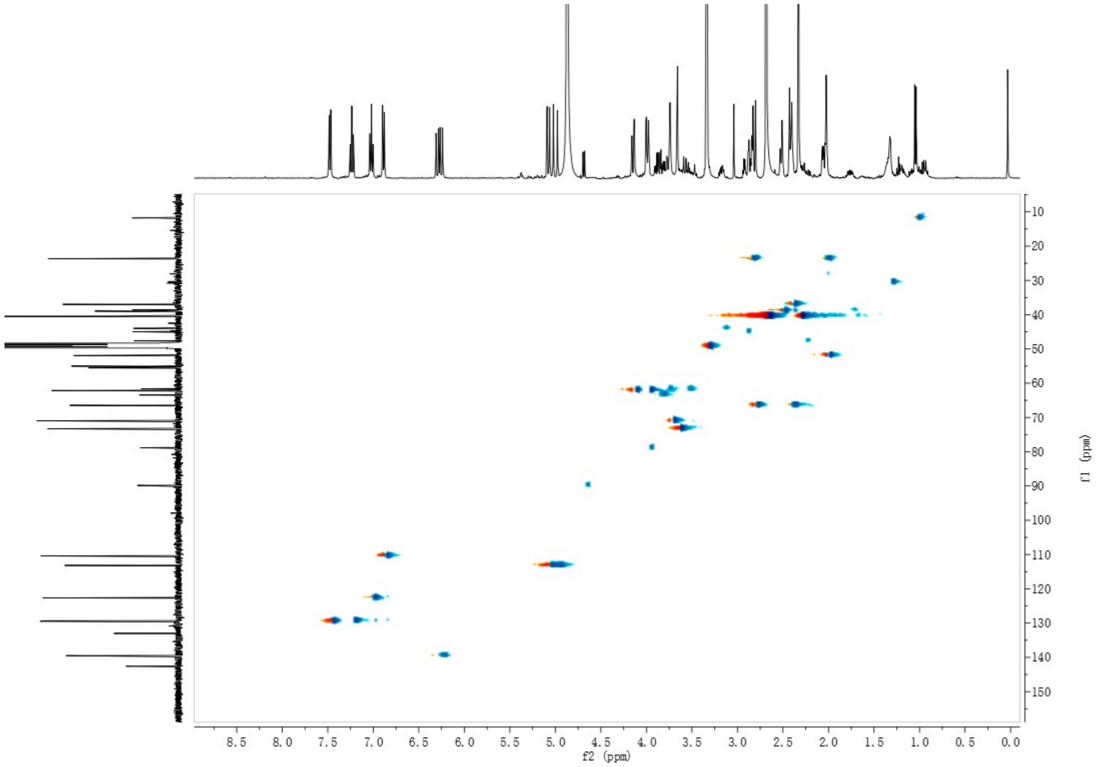

Figure S11. The HSQC spectrum of compound **2**.

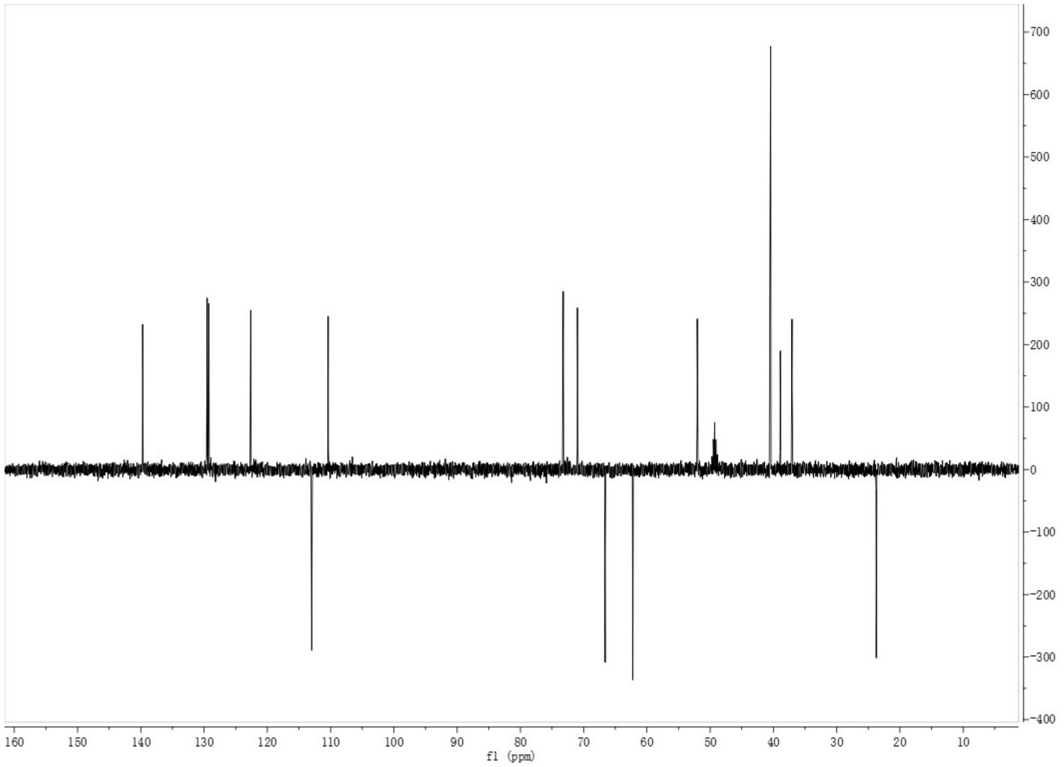

Figure S12. The DEPT135 spectrum of compound **2**.

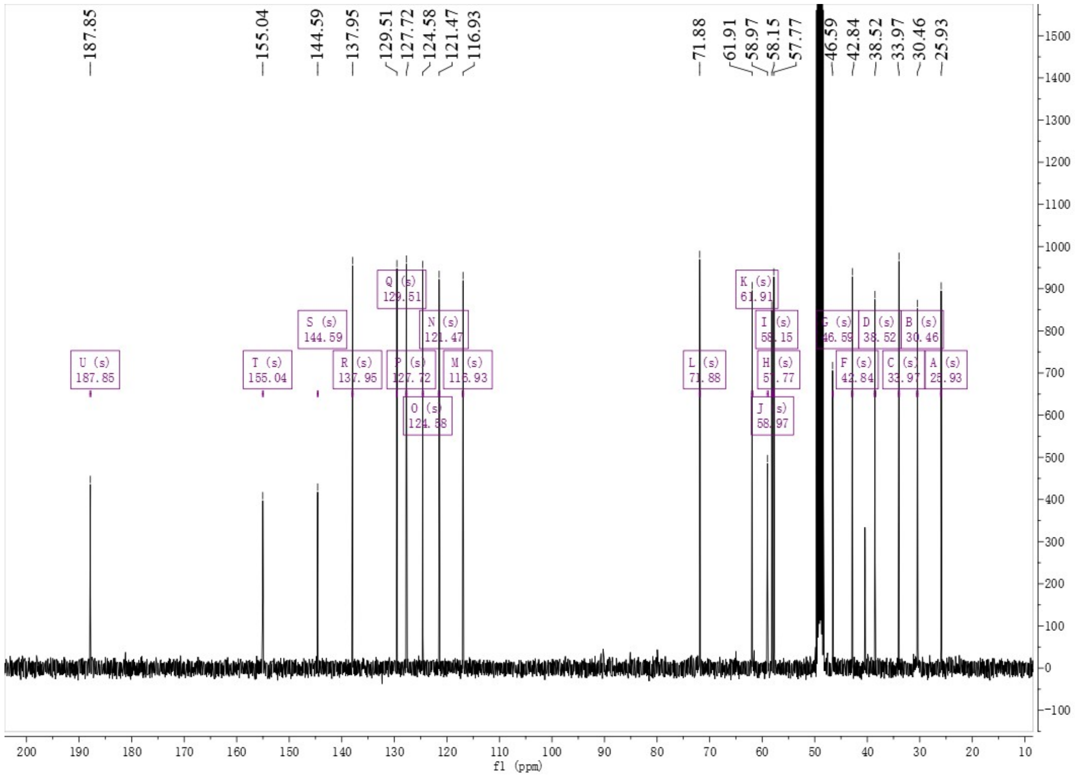

Figure S13. The ^{13}C NMR spectrum of compound **3**.

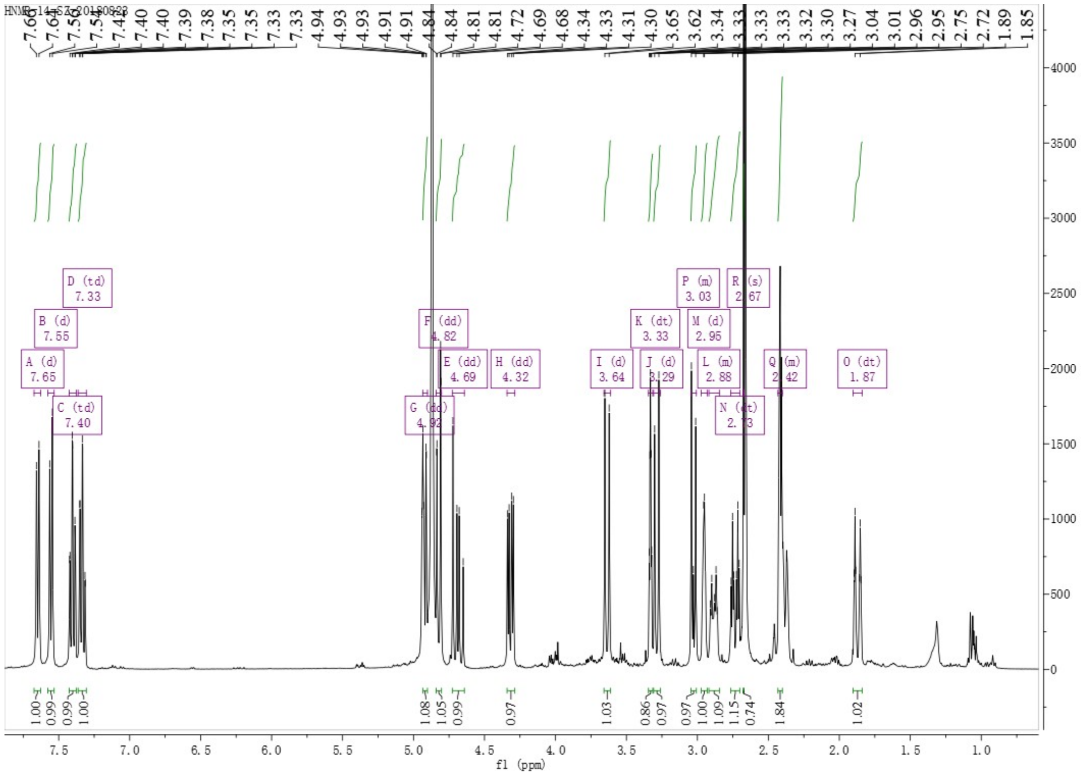

Figure S14. The ^{1}H NMR spectrum of compound **3**.
